# ELSAH (electronic smart patch system for wireless monitoring of molecular biomarkers for healthcare and wellbeing): definition of possible use cases

**DOI:** 10.3389/fbioe.2023.1166857

**Published:** 2023-05-04

**Authors:** Christian Brinkmann, Wilhelm Bloch, Giorgio C. Mutinati

**Affiliations:** ^1^ Department of Preventive and Rehabilitative Sport Medicine, Institute of Cardiovascular Research and Sport Medicine, German Sport University Cologne, Cologne, Germany; ^2^ Department of Fitness and Health, IST University of Applied Sciences, Düsseldorf, Germany; ^3^ AIT Austrian Institute of Technology GmbH, Center for Health and Bioresources, Molecular Diagnostics, Vienna, Austria

**Keywords:** continuous glucose monitoring, glucose, lactate, microneedle, patch, sensor

## Abstract

The ELSAH (electronic smart patch system for wireless monitoring of molecular biomarkers for healthcare and wellbeing) project has received funding from EU’s Horizon 2020 research and innovation program (grant agreement no. 825549). Its aim is to develop a wearable smart patch-based microneedle sensor system that can simultaneously measure several biomarkers in users’ dermal interstitial fluid. This system could have several use cases based on continuous glucose and lactate monitoring: early detection of (pre-) diabetes mellitus, increasing physical performance through optimal carbohydrate intake, achieving a healthier lifestyle through behavioral changes based on the interpretation of glucose data, performance diagnostics (lactate threshold test), control of optimal training intensities corresponding with certain lactate levels, or warning of diseases/health threats, such as the metabolic syndrome or sepsis associated with increased lactate levels. The ELSAH patch system has a high potential of increasing health and wellbeing in users.

## Introduction

Fueled by the growing functionality and affordability of miniaturized electronics, the widespread proliferation of smartphones and increasing consumer desire to monitor health data, wearables for health and wellbeing are on the rise (Heikenfeld et al., 2018). Currently available commercial products such as the *Fitbit* fitness tracker or the *Apple Watch* usually monitor several physiological variables and activities including heart rate, heart rate variability, or number of daily steps, which can also help predict maximum oxygen consumption or energy expenditure (Majumder et al., 2017).

An in-depth analysis of the user’s health, performance or stress level requires continuous quantification of molecular biomarkers which, of course, implies direct contact with the user’s biofluids (Heikenfeld et al., 2018). Blood is generally considered the gold reference biofluid to quantify molecular biomarkers. However, blood sampling is an invasive technique that isn’t compatible with the needs of wearable users. To that end, several wearables are currently being developed that use biofluids such as sweat (Sempionatto et al., 2017), saliva (Kim et al., 2014) or ocular fluid (Yao et al., 2011) to measure biomarkers, but these systems often face specific challenges linked to the respective non-invasive sample type. Among others, these challenges include poor correlations of biomarker concentrations to reference levels in blood, contamination or large fluctuations in pH that hamper reliable quantification of the target biomarkers (Mitsubayashi et al., 1994; Heikenfeld, 2016; Heikenfeld et al., 2018).

One way of dealing with this dilemma is to aim for minimally invasive sampling instead. The dermal interstitial fluid (ISF) has proven to be pH stable and highly comparable with the biomarker composition of blood (Tran et al., 2018). Furthermore, ISF can be sampled and analyzed using minimally invasive microneedles that have been rated as pain-free and are not susceptible to contamination (Sazonov, 2014; Ventrelli et al., 2015). Consequently, minimally invasive analysis of the ISF using microneedle-based sensors is the most promising approach for integrating continuous monitoring of biomarkers into wearables for healthcare and wellbeing.

**FIGURE 1:**
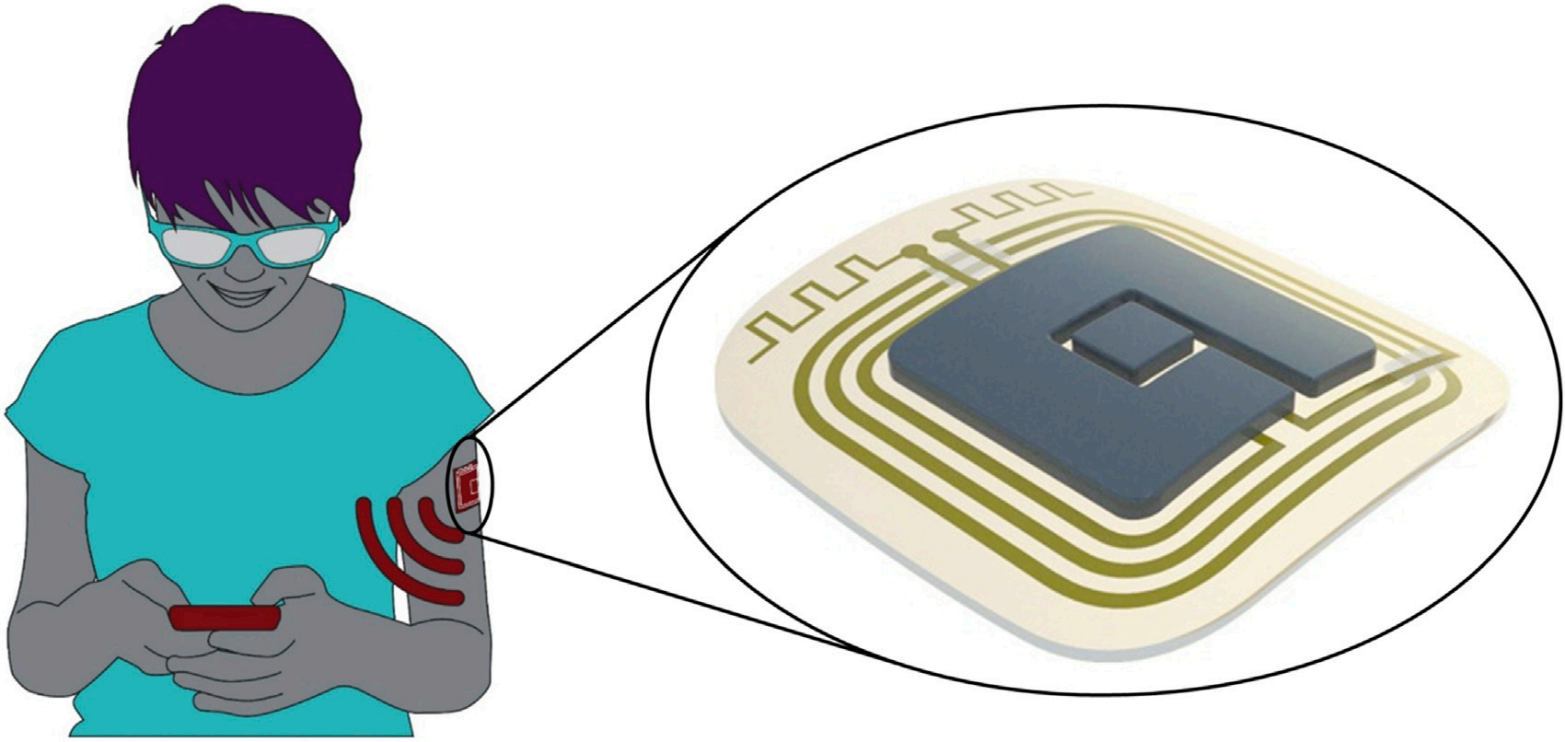
ELSAH consists of a smart patch-based microneedle sensor system that measures biomarkers in the user’s interstitial fluid and is connected to a mobile phone. Source and copyright holder: AIT/Purtscher and LEITAT (https://www.elsah.researchproject.at).

In the ELSAH project, a flexible and integrated smart patch-based wearable sensor system (“ELSAH patch”) is developed to quantitatively monitor several molecular biomarkers via electrochemical detection using a minimally invasive microneedle-based approach (Figure 1). The ELSAH patch will be fully self-sustained by integrating the microneedle biosensor, a microchip, a battery and antenna constructs into the patch, thereby enabling independent measurements and secure wireless data transmission to the user’s mobile phone.

In this article, based on the specifications of the ELSAH system, we will present possible applications of the ELSAH patch that we believe can increase health and fitness in users.

## ELSAH prototype and measurement technique

The final ELSAH patch will measure the two biomarkers glucose and lactate, which belong to the most reliable metabolic biomarkers to promote a healthy lifestyle (Goodwin et al., 2007; Klonoff et al., 2017). To comply with end user requirements for health and wellbeing monitoring systems (Holzer et al., 2020), the final ELSAH patch aims to feature the following properties:

1) Simultaneous electrochemical quantification of glucose and lactate concentrations in the ISF within a range of 0.3–30 mM;
2) Total monitoring period of 24 h at a measurement interval of 5 min;
3) Total patch area of 4 × 4 cm^2^ with a maximum patch thickness of 4 mm;
4) Choice of components and integration process resulting in manufacturing costs of a post-project commercial version of no more than EUR 3 per patch.

Various systems are available for determining glucose levels in diabetes care applications (continuous glucose monitoring (CGM) systems) (Rodbard, 2016). Most of them rely on probing the subcutaneous tissue ISF with the use of relatively large needles (5–13 mm in length) (Rodbard, 2016). These needles form electrodes that are functionalized by enzymes (usually glucose oxidase) which catalyze the electrochemical oxidation of glucose, thereby determining glucose concentration (Rodbard, 2016). Owing to the relatively large needles required for this system, it is not always in line with user needs, e.g., due to fear of needles (*trypanophobia*) and the possibility of subsequent bleeding/bruising (Yeoh et al., 2022). Furthermore, these systems are very expensive and are usually applied for long periods of time (1–2 weeks).

**FIGURE 2:**
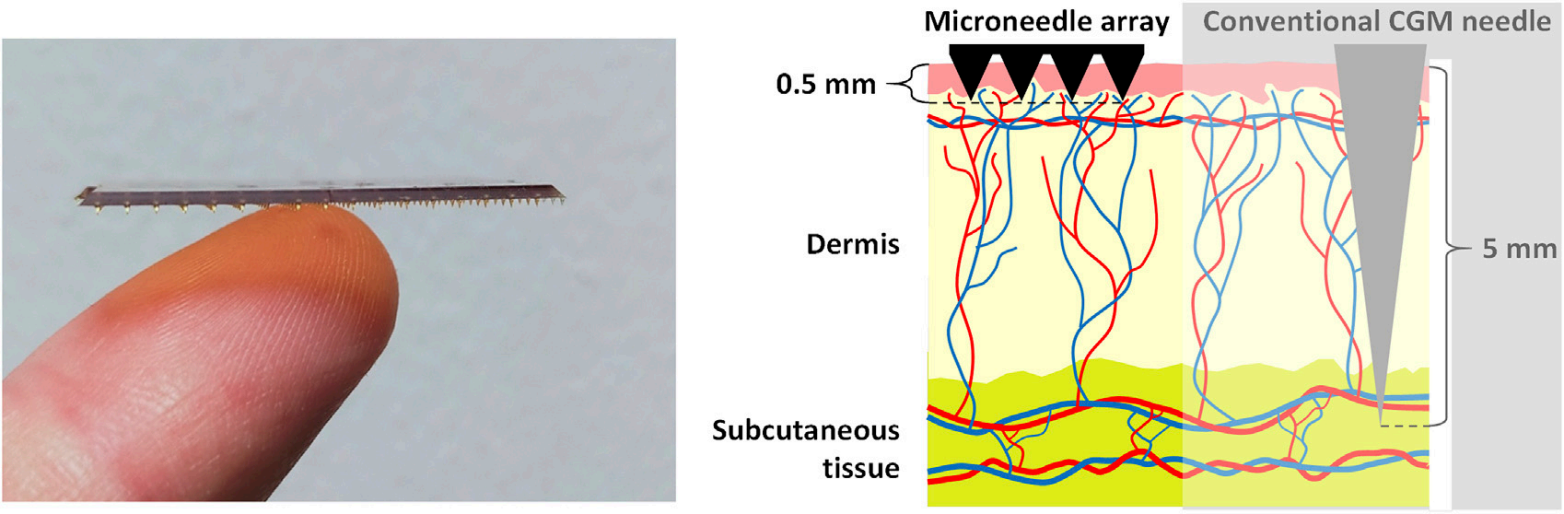
ELSAH polymeric microneedles (0.5 mm) compared to a human finger and a conventional CGM needle (≈5 mm) when inserted in the skin. Source and copyright holder: Tyndall National Institute and AIT (https://www.elsah.researchproject.at).

The microneedles used in the ELSAH system (with a microneedle length of approximately 0.5 mm) are pain-free to apply and wear (Ventrelli et al., 2015). Moreover, their production is compatible with mass manufacturing and is therefore inexpensive, they can be regularly exchanged and aren’t susceptible to contamination (Sazonov, 2014). The metallized polymeric microneedles in ELSAH are functionalized by a surface modification protocol involving direct electron transfer enzymes (cellobiose dehydrogenase-based), which enable the oxygen insensitive electrochemical detection of glucose and lactate (Jayakumar et al., 2022). For the quantification of glucose, a research group from the Imperial College London has already demonstrated in human test persons that a clinically acceptable correlation with the glucose level in blood can be obtained using microneedle-based sensing in ISF for an operating period of 24 h (Sharma et al., 2018). Furthermore, the study participants generally reported good tolerability of the microneedles with no long-term skin reactions, thus demonstrating good user comfort (Sharma et al., 2018). The ELSAH microneedles are shown in Figure 2.

## Possible use cases

Glucose is an important energy substrate for the brain, red blood cells, skeletal muscle and other cells and tissues. Hypoglycemia can lead to reduced brain and muscle function and, in the worst case, to hypoglycemic coma and death, while hyperglycemia can induce dehydration and life-threatening hyperglycemic coma (Wintergerst et al., 2006). It is also well-established that chronic increased blood glucose levels can cause cardiovascular complications in the long term (Stratton et al., 2000). Furthermore, hyperglycemia can alter lactate metabolism in the long term (Brinkmann and Brixius, 2015).

Lactate is released when pyruvate produced from glucose via anaerobic glycolysis is converted into lactate. Thus, it is indicative of the body’s metabolic state (Goodwin et al., 2007). Basal lactate levels can be increased in the context of several diseases/infections (e.g., diabetes mellitus, metabolic syndrome, cancer, sepsis) (Levraut et al., 1998; Wouters, 2002; Gustavsson et al., 2012; Brinkmann and Brixius, 2015; Mariappan et al., 2015; Oedorf et al., 2017; Spencer and Stanton, 2019).

### Use case 1: early detection of (pre-)diabetes mellitus

The metabolic state (“healthy,” “pre-diabetes” or “type 2 diabetes mellitus”) can be determined using glucose metrics. An early warning system for (pre-) diabetes mellitus could be established using the ELSAH patch. Common diagnostic methods include the fasting plasma glucose (FPG) test and the 75 g oral glucose tolerance test (OGTT) (Khan et al., 2019). According to the American Diabetes Association (ADA), FPG ≥100 mg/dL (5.5 mmol/L) or plasma glucose values 2 h post-75 g glucose ingestion ≥140 mg/dL (7.8 mmol/L) indicate pre-diabetes, while FPG ≥126 mg/dL (7.0 mmol/L) or plasma glucose values 2 h post-75 g glucose ingestion ≥200 mg/dL (11.1 mmol/L) indicate type 2 diabetes mellitus (ElSayed et al., 2023). The patch could be used to either measure fasting glucose values or glucose values during an OGTT in the ISF, with the ELSAH software providing the corresponding interpretation. In case of any indications of an abnormal glucose metabolism, the system could recommend the user to consult a medical doctor for further clarification. The ELSAH software could also evaluate several CGM-derived glycemic metrics such as “time in range” or glycemic variability over a 24-h period to provide a more detailed interpretation of the user’s glucose metabolism. Glucose variability has been discussed as a new option for determining glycemic control which is strongly correlated with vascular complications (Hirsch and Brownlee, 2005; Nalysnyk et al., 2010). However, CGM metrics are not yet well defined in subjects without diabetes, and additional studies are necessary to improve the accuracy of analyses that detect transitions from a healthy to a (pre-) diabetes state (Gottfried et al., 2022). The advantage of the ELSAH system diabetes test compared to others is that it is painless and bloodless. Furthermore, it is cheaper than having a blood sample taken by a medical doctor, which needs to then be analyzed in a laboratory. It is estimated that around 240 million people worldwide are currently living with undiagnosed diabetes (Ogurtsova et al., 2022). By using the ELSAH system for early detection of the disease, many patients could receive quick medical attention to slow down progression of the disease and prevent secondary complications.

### Use case 2: optimization of physical performance

Continuous glucose monitoring provides valuable information about athlete’s nutritional state before, during and post-exercise, and can thus influence decisions on how to improve physical performance (Holzer et al., 2022). There is evidence, for example, that the best times in long endurance runs are most likely achieved with only few high glucose fluctuations, and when a sufficient supply of carbohydrates is guaranteed (Oishi et al., 2018; Ishihara et al., 2020). The number of studies involving healthy subjects is very small; to be able to make more detailed recommendations, further studies in this field are urgently needed. The ELSAH software could define upper and lower limits, for example, and warn the athlete if he/she falls below or exceeds these limits. The athlete could, accordingly, increase or limit his/her carbohydrate consumption. The accuracy of personalized recommendations increases as more performance data are being synchronized with CGM data. The software could be further developed and synchronize data with a self-learning system. To our knowledge, such systems do not yet exist. The ELSAH patch allows for 24-h recording, and athletes can therefore use it as needed to monitor their glucose levels. This reduces costs compared to the use of conventional CGM systems.

### Use case 3: achieving a healthier lifestyle through behavioral changes based on the interpretation of glucose data

It has been shown that continuous glucose monitoring can help increase awareness of foods with high glycemic loads, thereby improving nutritional choices (Dehghani Zahedani et al., 2021). Continuous glucose monitoring could furthermore motivate people to increase their physical activity which is an effective way to decrease glucose values/postprandial glucose excursions (Liao et al., 2020; Bellini et al., 2021). The ELSAH system could potentially collect and display data on the individual’s “time in range.” The ADA’s glucose target range recommendation is 70–140 mg/dL (3.9–7.8 mmol/L) for patients with diabetes mellitus. A recent study based on CGM data of 4.805 individuals without diabetes mellitus indicates that a higher amount of time spent within an optimized glucose target range (70–100 mg/dL, 3.9–5.6 mmol/L) is associated with a more favorable cardiometabolic risk profile, while no such link was found for the time spent within the ADA’s target range (Berry et al., 2022). Long-term observational studies must be carried out to determine the actual risk of diseases and all-cause mortality in individuals without diabetes mellitus dependent on the different times they spend in different glucose ranges to answer the question whether stricter targets should be recommended for healthy subjects. ELSAH patches can be used as a cost-effective alternative to “classic” CGM systems for the collection of glucose data and can be used in a lifestyle intervention that motivates people to eat healthier or to more regularly be more physically active.

### Use case 4: performance diagnostics

Lactate level in blood is elevated (in most people up to ≈8–10 mM) following a progressive incremental exercise test to exhaustion, while the highest lactate levels (≈15–25 mM) are measured in the first few minutes after an “all-out” maximal exertion of 30–120 s (Withers et al., 1991; Goodwin et al., 2007). The ELSAH system could be used as a testing system with instructions for a step endurance test, the measurement of lactate levels and the calculation of individual lactate thresholds (lactate thresholds 1 and 2) beyond which lactate increases more rapidly. (Wackerhage et al., 2022). Lactate thresholds are considered well-established, relatively valid predictors of physical performance (Goodwin et al., 2007). A later increase in lactate levels during exercise testing indicates improved endurance performance (Goodwin et al., 2007). The ELSAH patch is expected to measure lactate more accurately than sensors that measure lactate in other biological fluids, such as sweat, saliva or ocular fluid, because lactate content is more variable in such fluids than in blood or in the ISF (Bollella et al., 2019). There is considerably interest in the development of highly sensitive lactate sensors that can measure lactate in real-time without blood sampling. Hence, lactate performance diagnostics could be carried out not only by sports medicine professionals, but by every coach or by the athlete himself/herself.

### Use case 5: determining and controlling training intensities

Exercise intensity is often prescribed by individual’s heart rate based on heart rate formulas which in many cases might be inaccurate (Goldberg et al., 1988). Knowledge of real-time lactate concentrations can be highly relevant for regulating training intensities during exercise as lactate levels adequately reflect the athlete’s metabolic state. (Goodwin et al., 2007). Based on individual performance diagnostics, training can be performed in different intensity ranges at, below or above the individual lactate thresholds. It is expected that training aligned with lactate thresholds provides a more homogenous training stimulus in a group of athletes than training that is based on heart rate formulas (Mann et al., 2013). However, more studies are needed to substantiate the theoretical advantages of threshold-based training programs (Jamnick et al., 2020).

### Use case 6: early warning of several diseases/health threats

Lactate can also be co-indicative for several diseases. Studies have shown increased fasting basal lactate levels (at rest), e.g., in patients with metabolic syndrome (≈+25%) (Jones et al., 2019). Basal lactate levels are also increased in the tumor environment in cancer patients (Mariappan et al., 2015; Spencer and Stanton, 2019). More studies are necessary to show the extent to which lactate might increase in neighboring areas in the ISF, and whether lactate in the ISF is a useful marker of tumor progression. Furthermore, lactate levels are associated with the degree of severity of acute infections and are drastically increased in septic patients (Levraut et al., 1998; Gustavsson et al., 2012; Oedorf et al., 2017). Basal lactate >4 mmol/L has been demonstrated to be an independent predictor of deterioration (e.g., acute renal failure, non-elective intubation, vasopressor administration or in-hospital mortality) for intensive care unit patients (Oedorf et al., 2017). Easily applicable continuous monitoring of lactate levels can open up entirely new perspectives for health-conscious end users. The ELSAH system could provide warnings when a certain lactate level is exceeded. The ELSAH system would then recommend to perform a health check by a medical doctor.

## Conclusion

The ELSAH patch system has a high potential of increasing health and wellbeing among users. Possible use cases have been presented for further development in the post-project phase. *In vivo* validation studies on measurement accuracy and further studies on possible lag times (blood→ISF), especially in the case of rapidly changing blood glucose or lactate values (during/post-exercise or meal intake), are needed (Madden et al., 2020). The ELSAH system will be fully self-sustained, allowing for independent measurements and secure wireless data transmission to the user’s mobile phone. Thereby, it assumes a pioneering role in the field of microneedle measuring systems. Microneedle-based technologies have immense potential for use in transdermal diagnostics, and a significant number of academic institutes and companies are developing highly miniaturized, patch-like sensors for biomarker monitoring (Madden et al., 2020; Tasca et al., 2019; Teymourian et al., 2021; biolinq, 2023; nutromics, 2023; sava, 2023). These alternatives to ELSAH are devices that are still being developed, and are manufactured using conventional Printed Circuit Board (PCB) technologies and plastic packaging methods. ELSAH aims to integrate the microneedle-biosensor and microchip into a fully disposable patch by using printed electronics technologies which will provide the antenna for wireless communication and the batteries for energy supply as well. ELSAH patches can be used by individuals who might need it only once in a while, as, for example, for the yearly medical check-up, without the need to invest in expensive electronic equipments, and is therefore affordable for any end user. One important aspect that will have to be addressed in the post-project phase is how to make the ELSAH patch fully environmentally sustainable. To this end, eco-design methodologies and medical device design processes must be incorporated into the future development.

## Data Availability

The original contributions presented in the study are included in the article/supplementary material, further inquiries can be directed to the corresponding author.
